# The fourth trimester and challenges for the lupus patient

**DOI:** 10.1016/j.ero.2025.10.009

**Published:** 2025-11-22

**Authors:** Erika G Fleming, Jamie Chen, Sarah Mohideen, Anna Broder, Antonia Francis Oladipo

**Affiliations:** 1 Hackensack Meridian School of Medicine, Nutley, NJ, USA; 2 Hackensack University Medical Center, Hackensack, Nutley, NJ, USA

## Abstract

Systemic lupus erythematosus (SLE) is a multisystem autoimmune disease that primarily affects persons of reproductive age. While SLE management during pregnancy has been previously studied, the unique challenges of the postpartum period, particularly the first 3 months after delivery, remain underexplored. The postpartum period is a time of significant physiological, social, and psychological change for both the birthing parent and infant. This review aims to summarise the available evidence regarding postpartum SLE flares, thrombosis, breastfeeding, perinatal microbiome, perinatal mood and anxiety disorders, maternal-infant bonding, and social support. Additionally, the review identifies significant knowledge gaps in postpartum SLE care and highlights priorities for future research to improve short-term and long-term outcomes for birthing parents with SLE and their infants.

## INTRODUCTION

To date, most studies related to pregnancy in patients with systemic lupus erythematosus (SLE) have focused on the 3 standard trimesters. However, the postpartum period, commonly referred to as the ‘fourth trimester’, has received significantly less attention [1]. This period, encompassing the first 12 weeks after delivery, presents unique challenges as both the birthing parent and infant adapt to their new environments. These challenges include SLE flares, increased risk of thrombosis, breastfeeding difficulties, the perinatal microbiome, perinatal mood and anxiety disorders, and maternal-infant bonding. These factors impact maternal and child health in both the short and long term. Due to the possible lifelong effect on maternal and paediatric health outcomes, optimisation and improvement of perinatal care are crucial. Thus, optimising postpartum care is essential, requiring a better understanding of existing research and the identification of gaps that future studies must address.

SLE affects an estimated 5.1 per 100,000 person-years, with women of reproductive age comprising the majority of those affected [2]. Therefore, understanding the relationship between the disease and complications of pregnancy is critical. PROMISSE (Predictors of Pregnancy Outcome: Biomarkers in Antiphospholipid Antibody Syndrome and Systemic Lupus Erythematosus), a landmark multicentre prospective study, was the first of its size and scope to investigate pregnancy outcomes in pregnant individuals with inactive, mild, or moderate SLE at the time of conception [3]. The study revealed that 19% of pregnancies resulted in adverse neonatal outcomes, including fetal death (4%), neonatal death (1%), preterm delivery (9%), and small-for-gestational age (10%). Davis-Porada et al [4] focused on the rate of flares within the PROMISSE cohort. Using the Safety of Estrogens in Lupus National Assessment-Systemic Lupus Erythematosus Disease Activity Index Flare Index, they found that participants were more likely to have mild than severe intrapartum flares (20.8% vs 6.25%). The same trend was observed for postpartum flares (27.7% vs 1.7%). Both intrapartum and postpartum flares rarely required additional treatment past mild increases in glucocorticoid doses [4]. While pioneering studies like PROMISSE advanced understanding of antenatal SLE activity, they also highlighted the need for further exploration of postpartum-specific issues.

The postpartum period introduces unique challenges for SLE patients that remain underexplored, including (1) heightened risk of flares, (2) thrombosis, (3) medication concerns during breastfeeding, (4) understanding the maternal-fetal dyad and neonatal microbiome, (5) heightened risk of postpartum depression, and (6) social and bonding issues. This review aims to summarise existing information and identify gaps for future research (Table).

**Table:** Summary of key findings, knowledge gaps, and recommendations for patients with systemic lupus erythematosus in the fourth trimester

Understudied areas postpartum	Key findings	Research gaps and future areas of focus	Key takeaways
Disease flares	• The postpartum period carries a heightened risk of lupus flares 6-12 months after delivery in the setting of immunological changes. • Disease activity at conception is correlated with increased rates of postpartum flares. • Medication nonadherence is a major driver of postpartum flares.	• Medication adherence patterns and reasons for discontinuation in postpartum women with SLE • Role of immunological shifts in postpartum flares • Targeted interventions for medication adherence in postpartum SLE patients	• Maintain disease control from conception and into postpartum with continuation of essential therapies, with adjustment for breastfeeding preferences. • Strengthen interdisciplinary collaboration among physicians caring for the postpartum SLE patient. • Implement multifaceted and targeted strategies for postpartum medication adherence.
Thromboembolism	• Postpartum is the highest risk window for thromboembolism. • SLE increases thrombotic risk through systemic inflammation, increased platelet activation, and endothelial dysfunction. • Pregnancy complications in SLE pregnancies further amplify risk. • Prevention strategies for thromboembolism are underutilised and insufficiently tailored.	• SLE-specific risk assessment models for thromboembolism in the postpartum setting • Optimal prophylactic anticoagulation strategies tailored to postpartum SLE patients • Systemic barriers to postpartum thromboprophylaxis in SLE	• Implement individualised risk assessment incorporating SLE-specific factors, such as antiphospholipid antibody status, lupus nephritis, and prior thrombosis. • Provide prophylactic anticoagulation for high-risk postpartum SLE patients, guided by interdisciplinary teams. • Educate patients on venous thromboembolism risk, early symptom recognition, and adherence to preventative therapies.
Breastfeeding	• Despite its numerous benefits, breastfeeding rates are lower among postpartum women with SLE. • Medication concerns are a leading barrier to breastfeeding. • Breastfeeding rates vary by race and maternal disease activity. • Education and structured support can improve breastfeeding adherence and duration.	• Effective interventions to better support breastfeeding among SLE patients • Further studies on medication safety, especially in newer biologics	• Education for patients and providers alike on safe lupus medications during breastfeeding. • Expand lactation support programmes tailored for mothers with SLE. • Address inequalities by targeting high-risk groups and integrating disease management with breastfeeding counselling.
Microbiome	• The perinatal microbiome is critical to infant health, with breastfeeding playing a direct role in microbiome transmission. • Lupus is associated with microbiome dysbiosis. • Maternal microbiome alterations in SLE may influence neonatal microbiome development.	• The effect of maternal SLE-associated microbiome dysbiosis on infant microbiome development and long-term immunological outcomes • Lupus medication during pregnancy and lactation on both maternal and neonatal microbiome composition • Studies examining the microbial composition of breast milk in SLE patients and how it differs from healthy controls	• Encourage breastfeeding when medically appropriate, given its role in establishing a healthy infant microbiome. • Discuss microbiome-preserving practices (limiting unnecessary antibiotic use, promoting vaginal delivery when obstetrically safe) to help optimise maternal and neonatal microbiome development. • Assess and monitor disease activity closely in the postpartum period, as high disease activity is associated with greater dysbiosis.
Postpartum depression	• Postpartum women with SLE are at greater risk of PPD, due to higher baseline prevalence of major depressive disorder, disease burden, and social support. • Immunological disturbances in lupus may contribute to the development of PPD among other mood disorders.	• Prevalence, severity, and clinical course of PPD within the SLE population • Biologic mechanisms linking postpartum immune dysregulation and depression in SLE • Longitudinal studies assessing how PPD impacts long-term lupus disease course, maternal outcomes, and infant development	• Implement routine, standardised PPD screening for all postpartum patients with lupus, beginning in prenatal care and continuing through the postpartum period. • Improve access to evidence-based treatment options for postpartum SLE patients.
Bonding and social support	• The postpartum period is critical for birthing parent-infant bonding, which supports the infant’s emotional, social, and cognitive development. • Patients with SLE are at higher risk for psychological distress due to compounding factors. • Social support is a strong protective factor against postpartum depression and anxiety and is associated with better maternal and infant outcomes.	• The longitudinal impacts of SLE on parent-infant bonding in the postpartum period • Effectiveness of social support interventions for postpartum SLE patients • Effects of racial, socioeconomic, and healthcare disparities and their intersection with lupus on postpartum mental health and bonding outcomes	• Assess and strengthen social support in postpartum SLE patients, including partner, family, and community resources using culturally sensitive approaches. • Social prescribing, connect SLE postpartum patients in need with available support services.

PPD, postpartum depression; SLE, systemic lupus erythematosus.

This table provides an overview of the current evidence and remaining knowledge gaps regarding common challenges faced by patients with SLE in the postpartum period (‘the fourth trimester’). It summarises findings related to disease flares, thromboembolism, breastfeeding considerations, microbiome changes, postpartum depression, and social support, along with corresponding recommendations and strategies for management.

## THE FOURTH TRIMESTER AND LUPUS FLARES

### Increased risk of flares in the postpartum period

Flares are thought to be triggered by a combination of environmental and physiological factors, including medications, infectious illness, injury, and emotional stress [[5], [6], [7]]. The physical and emotional demands of the antenatal period, including labour, delivery, and caring for a newborn, add significant stress during the fourth trimester [8], emphasising the importance of understanding its relationship to postpartum flares. Reported rates of mild to moderate lupus flares during the postpartum period vary widely, ranging from 27.7% to 41% in different studies [4,9].

A study of 145 Norwegian pregnant women with SLE found that most experienced minimal disease activity during conception and pregnancy. However, disease flares increased at 6 months (2.8%) and 12 months (5.1%) postpartum [10]. Similarly, Ntali et al [11], in a prospective study of 82 southern European pregnant patients with SLE, observed a higher incidence of flares postpartum, with rates of severe flares more than twofold higher compared to the gestational period, particularly among those with alopecia, hypocomplementemia, and nephritis. Postpartum immunologic changes may explain this trend [12]. Björkander et al [13] measured chemokine levels in pregnant women with and without SLE during the second and third trimesters and 8-12 weeks postpartum. Women with SLE consistently showed higher levels of CXCL8/IL-8, CXCL9/MIG, CXCL10/IP-10, and IL-10, particularly in those with greater disease activity. These chemokines are believed to drive inflammatory pathways that contribute to postpartum flares. Of particular interest is IL-10. Although recognised as an anti-inflammatory cytokine, it is hypothesised to serve as a primary driver of autoantibody production in SLE [14,15]. Additional factors that predict postpartum flares include advanced maternal age and increased disease or serological activity at conception [16]. Radin et al [17], which followed pregnant women with SLE for up to 2 years postpartum, found that patients with remission at conception had lower rates of flares, even in the long term.

### Lupus flares and medication adherence

Medication nonadherence is a key factor contributing to SLE flares in the postpartum period. Rates of nonadherence among SLE patients overall vary widely, ranging from 12% to 98%, due to inconsistent definitions and assessment methods [[18], [19], [20]]. Medication nonadherence is particularly pronounced during pregnancy and the postpartum period [21]. A Canadian population-based study of 376 SLE pregnancies found a significant decline in the use of antimalarials, azathioprine (AZA), and glucocorticosteroids during and after pregnancy. For example, only 31.1% of women with SLE continued antimalarials postdelivery compared to 36.2% prepregnancy. Similar decreases were observed for AZA (11.7% to 10.4%) and glucocorticosteroids (33.2% to 25.3%) [21].

The association between the discontinuation of immunosuppressive medications postpartum and increased SLE flares is well documented [10,16,22]. Götestam-Skorpen et al [10] reported higher disease activity at 6 months (2.8%) and 12 months (5.1%) postpartum compared to conception and pregnancy. While most SLE patients in the study remained adherent to hydroxychloroquine (HCQ) and prednisolone, AZA use decreased from 30% in the first trimester to 19.6% at 12 months postpartum, with most discontinuations occurring by the third trimester or 6 weeks postpartum. No subsequent initiation of other immunosuppressive medications was observed. Notably, 80% of women who stopped AZA reported breastfeeding, though the study did not explore their reasons for discontinuation. Similar patterns were observed in the Ntali cohort, where the use of HCQ and AZA declined during the puerperium, hypothesised to reflect concerns about drug safety while breastfeeding. This discontinuation paralleled the rise in both frequency and severity of SLE flares observed during the second and third postpartum trimesters [11]. Eudy et al [22] also found that postpartum flares were more common in patients who discontinued HCQ during pregnancy compared to those who continued it. While Eudy et al [22] and Ntali et al [11] used a longitudinal cohort to track disease activity over time, Götestam-Skorpen et al [10] relied on patient-reported outcomes, providing complementary perspectives on HCQ adherence. None of the aforementioned studies investigated the reasons for the discontinuation of medication.

Shaharir et al [16] conducted a univariate analysis in Malaysia, emphasising that consistent HCQ use—beginning 3 months before pregnancy and continuing throughout—significantly reduced postpartum flares in SLE. Similarly, studies like Eudy et al [22] demonstrate that adherence to broader SLE immunosuppressive therapies, such as AZA and HCQ, helps minimise postpartum disease activity. Together, these findings highlight the critical importance of maintaining immunosuppressive SLE treatments throughout pregnancy and the postpartum period. The study by Eudy et al [22] is particularly notable for directly comparing outcomes based on HCQ continuation vs discontinuation during pregnancy in individuals with SLE.

### Strategies to improve medication use

Medication nonadherence in individuals with SLE is influenced by multifactorial and complex factors. Predictors of nonadherence include younger age at diagnosis, nonuse of steroids, higher body mass index (BMI), and unemployment [23]. Depression and poor prognostic factors have also been identified as major contributors, while factors such as income, health insurance, disease duration, and health literacy appear to be less influential [24]. Conversely, protective factors include resilience, older age, improved quality of life, and supportive medical relationships [25,26].

Pregnant patients with SLE are advised to work closely with their physicians to develop a postpartum treatment plan that minimises the risk of disease flare, with particular attention to maternal preferences regarding breastfeeding when selecting medications [27]. Physician perspectives underscore 2 main barriers to medication adherence among pregnant patients with SLE: limited patient trust in provider recommendations and gaps in clinician knowledge about appropriate medications [28]. Additionally, poor communication between rheumatologists and obstetrician-gynaecologists (OBGYNs) emerged as a concern, often leading to inconsistent treatment plans and patient confusion. Regional differences further highlight the importance of effective interdisciplinary collaboration. For instance, in Northern California, where rheumatologists and OBGYNs reported stronger connections, medication adherence rates among pregnant SLE patients were notably higher (83.6%-93.2% medication fill rate) [28,29]. These findings suggest that strengthening interdisciplinary communication is a key strategy for improving adherence, particularly during the postpartum period.

Accurate assessment of medication adherence in patients with SLE is crucial, as misevaluation can result in unnecessary treatment escalation and misinterpretation of nonadherence as therapeutic failure [19,30]. Several tools have been utilised to assess medication adherence in patients with SLE, with varying degrees of effectiveness. Blood level measurement, particularly for HCQ, provides a simple, objective, and reliable indicator of adherence [19,31]. Discussing low levels with SLE patients has been shown to initiate open dialogue and foster better adherence [32]. Other methods, such as medication event monitoring systems, pharmacy records, healthcare provider assessments, and self-reported questionnaires, have also been explored, though they vary in efficacy among the broader SLE patient population [33].

To date, no single intervention has proven universally effective in improving adherence among SLE patients [19,34,35]. Rather, a combination of strategies is required to address the multifaceted factors influencing nonadherence, particularly in the postpartum period. These strategies include (1) addressing underlying predictors of nonadherence, such as depression and unemployment, (2) fostering supportive and trusting medical relationships, (3) encouraging patient involvement in medication decisions, (4) strengthening interdisciplinary communication between rheumatologists and OBGYNs, and (5) screening for nonadherence through reliable tools like blood level measurements. Future research should focus on postpartum adherence rates and explore targeted interventions to improve medication use during the fourth trimester.

## THROMBOEMBOLISM IN THE FOURTH TRIMESTER LUPUS PATIENT

### Increased risk of thromboembolism

The postpartum period represents the highest risk for thromboembolism in the perinatal period. Postpartum individuals face an elevated risk of venous thromboembolism (VTE), deep venous thrombosis (DVT), and pulmonary embolism, largely attributed to Virchow’s triad—a framework for identifying factors contributing to thrombosis [36]. During labour and delivery, birthing parents may experience vascular injury as well as venous stasis from decreased mobility during recovery [37]. The hypercoagulable state of pregnancy—characterised by increased clotting factor production and resistance to Protein C and S—further amplifies this risk [36]. Postpartum individuals are 15- to 35-fold more likely to experience thromboembolism compared to nonpregnant individuals, with the risk peaking in the first 3–6 weeks and extending up to 12 weeks after delivery [[38], [39], [40], [41]].

Patients with SLE are uniquely predisposed to thromboembolism due to inflammation and autoimmune factors [42]. Chronic inflammation impacts multiple steps in coagulation, reducing fibrinolytic activity, increasing plasminogen activator inhibitor production, and impairing the Protein C pathway [43]. In addition, antiphospholipid antibodies, commonly found in SLE patients, promote a prothrombotic state by binding plasma proteins and triggering platelet activation, increasing the risk of both arterial and venous thrombosis [[44], [45], [46]]. Proteinuria from lupus nephritis further exacerbates clotting abnormalities, raising the risk of DVT and renal vein thrombosis [47].

Postpartum patients with SLE face compounded risks due to the overlapping factors of postpartum physiology and SLE pathophysiology. Pregnancy complications, such as preeclampsia, hypertension, and caesarean delivery, more common in SLE patients, further heighten this risk [37,48,49]. A 2023 retrospective cohort study revealed that postpartum patients with SLE are 2.5 times more likely to develop VTE, with lifetime thrombosis rates of 10% to 26% in this population [50]. Additionally, rare complications such as ovarian vein thrombosis, phlegmasia cerulea dolens, and thrombotic microangiopathy have been reported in postpartum SLE patients [51,52].

### Thromboembolism prevention strategies

Risk stratification and tailored prophylactic management should be the standard of care for postpartum SLE patients. However, current practices often fall short. A cohort analysis found that although over one-third of pregnant SLE patients were classified as high-risk based on Royal College of Obstetricians and Gynaecologists guidelines, only 19% received appropriate thromboprophylaxis [53].

Preventive strategies should address both general postpartum thromboembolism risks and SLE-specific factors [54]. While risk assessment models (RAMs) exist for the general obstetric population, none are tailored specifically for patients with SLE, highlighting the need for more precise tools [36]. Overall, there is a need for more tailored care to meet the specific needs and concerns of postpartum patients with a history of lupus. Clinicians should emphasise (1) individualised risk assessment: incorporating factors such as antiphospholipid antibody status, lupus nephritis, and history of thrombosis; (2) prophylactic anticoagulation: for high-risk patients, considering low–molecular-weight heparin or other anticoagulants based on clinical guidelines; (3) interdisciplinary care: enhancing collaboration between rheumatologists, obstetricians, and haematologists to ensure consistent and effective management plans; and (4) patient education: improving patient understanding of thrombosis risks, symptoms, and the importance of adherence to preventive therapies. Future research should focus on developing SLE-specific RAMs, optimising anticoagulation strategies, and identifying systemic barriers to postpartum thromboprophylaxis. Addressing these gaps will be critical to improving outcomes for postpartum SLE patients.

## FACTORS INFLUENCING BREASTFEEDING IN LUPUS PATIENTS

### Advantages of breastfeeding

Breastfeeding plays a critical role in the health and development of both the breastfeeding parent and neonate. Breast milk provides complete nutrition and promotes early immunity through vital immunoglobulins and microbiota for the newborn [[55], [56], [57]]. It also fosters emotional bonds, supports infant social-emotional development, and reduces maternal stress [58]. Long-term benefits for infants include reduced risks of asthma, allergies, type 1 diabetes, and obesity, while breastfeeding parents experience favourable metabolic changes that reduce their risk of chronic diseases [59]. Given these advantages, major health organisations like the American Academy of Pediatrics, World Health Organization, and United Nations Children’s Fund recommend exclusive breastfeeding for the first 6 months and continued breastfeeding alongside other foods for up to 2 years.

### Contributing factors for decreased breastfeeding rates in lupus patients

Postpartum SLE patients face unique challenges that may hinder breastfeeding. A study by Noviani et al [60] tracking 51 pregnancies in individuals with SLE found that although 67% of women intended to breastfeed, only 49% successfully did. Factors such as concerns about medication (42%), low milk production (27%), and difficulties coping (23%) were cited by those who discontinued breastfeeding. African American patients had lower rates of both breastfeeding intent (55% vs 78%) and actual breastfeeding (35% vs 57%), as well as higher postpartum lupus activity compared to their Caucasian counterparts [60].

In a similar study by Orefice et al [61], postpartum patients with SLE were found to have an initial breastfeeding rate similar to the general population, with 96.5% intending to breastfeed; 71.9% of women within the study initially breastfed, but 50% discontinued within 3 months, higher than the 30% seen in healthy women. Contributing factors included insufficient milk, prematurity, medication use, difficulty latching, smoking, higher BMI, and joint involvement related to disease activity. Interestingly, extended disease duration and HCQ treatment were linked to longer breastfeeding durations, likely due to its efficacy in reducing disease activity [61].

Acevedo et al [62] also found that individuals with SLE were more likely than individuals without SLE to never initiate breastfeeding (19.4% vs 5.6%) and had shorter breastfeeding durations (an average of 6 months vs 12 months). Interestingly, 41% of participants with SLE in the study cited medication as a reason for early weaning. Despite concerns, many medications used to treat lupus, including low-dose corticosteroids and HCQ, are considered safe during breastfeeding. This underscores the importance of addressing inadequate education about safe medications for breastfeeding.

### Breastfeeding and medication safety

Concern about medication use is a major barrier to breastfeeding among SLE patients. However, the American College of Rheumatology guidelines (2020) strongly encourage breastfeeding, as most SLE medications are considered safe [63]. Medications like ibuprofen, hydroxychloroquine, colchicine, tumour necrosis factor (TNF) inhibitors, and rituximab are safe for breastfeeding without dosage adjustments. Other biologics, such as anakinra, abatacept, tocilizumab, secukinumab, and ustekinumab, are presumed to have minimal transfer through breast milk due to their large molecular size, though data on relative infant doses are limited. AZA is considered reasonably safe to continue during breastfeeding [64]. Corticosteroids, which are commonly used to control lupus flares, are recommended to be taken 4 or more hours before breastfeeding for doses of more than 20 mg. Mycophenolate, cyclophosphamide, and thalidomide are contraindicated during breastfeeding [63].

### Strategies to improve breastfeeding adherence

Given that most first- and second-line lupus medications are compatible with breastfeeding, healthcare providers should encourage patients with SLE to breastfeed according to established guidelines [63,65]. Educating paediatricians, obstetricians, and lactation consultants on the safety of SLE medications during breastfeeding could help alleviate concerns and improve breastfeeding rates. Additionally, interventions aimed at increasing breastfeeding self-efficacy—such as lactation support programmes, educational materials, breastfeeding room availability, and access to breastfeeding consultants—may be effective in addressing the challenges faced by SLE patients. Further research is needed to identify effective interventions and better understand the barriers to breastfeeding in this population.

## THE RELATIONSHIP BETWEEN LUPUS AND THE PERINATAL MICROBIOME

### Understanding the perinatal microbiome

The human microbiome, an intricate ecosystem composed of bacteria, viruses, fungi, and other microorganisms residing within and on the human body, has emerged as a field of considerable interest. The interactions among these microorganisms, which can be commensalistic, mutualistic, or even pathogenic, play a critical role in overall health and the body’s response to environmental factors [66]. Dysbiosis, or imbalances in the microbiome, has been linked to various diseases, including autoimmune conditions [67]. Emerging research challenges the long-held belief that the fetal environment is sterile, revealing that bacteria are present in the placenta, umbilical blood, and meconium [[68], [69], [70]]. These findings suggest maternal transmission of microbiota, which may shape the neonate’s immune system. Early microbiome development continues after delivery, with significant shifts until the age of 3, at which point the gut microbiome stabilises [71]. Factors like diet, gestational age at birth, and antibiotic use influence this maturation [72]. The gut microbiome is crucial for immune function, digestion, and pathogen resistance [73]. Proper development of the neonatal microbiome is linked to lower risks of asthma, allergies, and autoimmune diseases [74]. Breast milk directly shapes the infant microbiome not only by influencing floral composition through nutrient selection but also by introducing bacterial species directly into the infant’s gastrointestinal tract [75]. In fact, approximately 88% of the genera are common between breast milk and the stool of breastfed infants, further supporting direct transmission from the birthing parent [76].

### Understanding lupus and the microbiome

To understand the neonatal microbiome, we must first explore the microbiome of the birthing parent with SLE. Studies suggest that alterations in the gut microbiome are key in lupus pathogenesis, potentially driving autoimmunity and inflammation through alterations in the innate and adaptive immune system [77]. Research shows that SLE patients have reduced microbiota richness and diversity compared to healthy individuals, with more pronounced reductions in those with high SLE disease activity [78,79]. Specific microbiota, such as *Lactobacillaceae* and *Lachnospiraceae,* are linked to disease activity and severity in lupus, both in animal models and human patients [78,80]. Transfer studies have further demonstrated the causal effect of the microbiota on SLE. In faecal transfer from SLE mice to germ-free mice, recipient mice developed anti-dsDNA antibodies and showed upregulated expression of SLE susceptibility genes. Additionally, faecal transfer was associated with altered distribution of immune cells in mucosal and peripheral immune responses, further supporting the microbiome’s role in lupus pathogenesis [81]. This strong background of research into the microbiome in the setting of SLE provides a framework for understanding the impact on the infant of a patient affected by the disease.

### The bidirectional impact of lupus and the microbiome in the postpartum period

It is well established that the infant’s microbiome is profoundly influenced by several key factors, including passage through the vaginal birth canal and breastfeeding. However, the specific influence of an SLE-affected birthing parent on the neonatal microbiome remains understudied and raises important questions. How do lupus-associated changes in the maternal microbiome affect the infant’s microbiome development? Does breastfeeding in SLE birthing parents alter microbial transmission compared to their healthy counterparts? Furthermore, how do lupus medications impact maternal and infant microbiomes during lactation? Addressing these gaps through advanced techniques, such as metagenomics, is key to understanding how the maternal microbiome impacts infant health. This knowledge could potentially lead to the development of strategies aimed at improving outcomes for SLE-affected patients and their children. Research on the composition of breast milk in lupus patients and its influence on the infant microbiome could provide new insights into postpartum health and disease prevention.

## POSTPARTUM DEPRESSION IN THE LUPUS PATIENT

### Overview of postpartum depression

As highlighted in the previous sections on flares, breastfeeding, and thrombosis, there are a plethora of rapid physiologic changes that the body undergoes in the postpartum period. These changes can greatly impact mood and behaviour. Feelings of sadness or emptiness are common for the first few days after giving birth, most likely due to the sudden drop in oestrogen and progesterone levels after delivery [82]. These symptoms, often referred to as ‘baby blues’, typically recede within 3-5 days [83].

However, symptoms that last longer than 2 weeks are concerning for postpartum depression (PPD). PPD is believed to affect between 6.5% and 12.9% of the general population [84]. In contrast to the heightened emotions and sadness seen in postpartum blues, PPD is characterised by feelings of emptiness and anhedonia, similar to symptoms of major depressive disorder (MDD) [84]. This can hinder daily activities and make bonding with the newborn more difficult. Postpartum depression can also jeopardise the infant’s sense of security with their primary caregiver, possibly increasing vulnerability to abnormal brain development and dysregulated sleep [85].

### Increased risk of postpartum depression in the lupus patient

Patients with SLE are at increased risk of PPD due to a combination of factors. Previous episodes of MDD are a risk factor for PPD [86,87]. Lupus patients have a significantly higher prevalence of MDD, with up to 24% of individuals diagnosed, compared to just 3.8% in the general population [88,89]. Factors such as disease burden, fatigue, poor sleep quality, and a lack of psychosocial support contribute to this increased risk [90,91].

Secondly, the immunologic disturbances present in lupus, including chronic inflammation through TNF-receptor transcription and neutrophil-mediated immunity, may further predispose lupus patients to mood disorders such as PPD [92]. Lin et al [93] followed 45,451 individuals in the postpartum setting from 1996 to 2013 and found that patients with PPD have higher risks of subsequent autoimmune diseases, especially pernicious anaemia, rheumatoid arthritis, and Graves’ disease. While the study did not investigate SLE, the research findings of both Mehta et al [92] and Lin et al [93] call for further investigation into the relationship between immunologic changes in both PPD in SLE patients and its effect on the disease course. Skinner-Taylor et al [94] found that in an observational, cross-sectional, and descriptive study of women with autoimmune rheumatic diseases, women with SLE had the highest mean score on a PPD questionnaire. However, studies specifically investigating PPD in SLE patients are limited, and more research is necessary to further understand the extent to which mothers with SLE are at increased risk for PPD.

In addition, patients with lupus face unique challenges in the postpartum period, such as the need to adjust medications and manage potential disease flares, which can contribute to both emotional and physical strain. The hormonal and immune system changes that accompany childbirth may exacerbate underlying depression and anxiety in lupus patients, creating a cycle that further impairs postpartum mental health. However, the specific impact of lupus on PPD has been understudied, and more research is needed to understand the full extent of this connection.

### Strategies to increase screening and treatment for PPD

Providers can employ a multifaceted approach to enhance the recognition, diagnosis, and treatment of PPD. Routine screening for PPD is essential for all postpartum parents, especially those with lupus, who may experience more pronounced symptoms. Standardised tools like the Edinburgh Postnatal Depression Scale should be used during prenatal and postpartum visits to facilitate early detection [95,96]. Once identified, comprehensive treatment options—including cognitive-behavioural therapy and selective serotonin reuptake inhibitors—should be offered to manage PPD symptoms effectively [97,98]. The first oral medication indicated specifically for PPD, zuranolone, a positive allosteric modulator of GABA-A receptors, has recently been approved by the Food and Drug Administration and shows promise as a future mainstay treatment for PPD [99]. Finally, education about PPD is imperative as it encourages help-seeking behaviour as well as early symptom recognition [100].

In summary, a systematic approach that encompasses screening, education, targeted interventions, and ongoing monitoring is essential for effectively addressing PPD and promoting both maternal and neonatal health.

## THE SOCIAL ASPECT OF THE FOURTH TRIMESTER

### Importance of parent-infant bonding in the fourth trimester

Postpartum marks the beginning of the parent-infant relationship, a period of great importance for the development of the infant. Birthing parent-infant bonding is foundational to the infant’s emotional and social development, with secure attachment linked to better prosocial behaviour and cognitive outcomes [101]. However, maternal psychological distress, including PPD, is associated with impaired self-reported bonding [102]. Lupus patients, who may face compounded psychological and physical stressors, are particularly at risk. While preliminary research [103] in a small study of 15 third-trimester Italian women with various autoimmune diseases, with 1 patient having SLE, suggests that autoimmune disease may not universally impair bonding, larger studies focused on postpartum SLE patients are needed to clarify this relationship.

### The importance of social support during the postpartum period

Given that patients with lupus may have a higher risk of PPD, healthcare providers must be cognizant of the increased need for social support in SLE patients entering the postpartum setting. Social support has a protective effect against PPD and postpartum anxiety, improving both maternal and neonatal outcomes [104]. Emerging research has demonstrated that low social support is associated with a 4.63-fold increase in developing PPD [105]. Limiting anxiety is of equal importance, as postpartum anxiety is associated with lower levels of self-confidence and increased fatigue in postpartum individuals, as well as sleep disturbances, excessive crying, poor social engagement, and poor cognitive and motor development in infants [104].

Social support encompasses emotional and informational assistance from partners, family, healthcare providers, and communities. Support includes cognitive-behavioural counselling, nondirective counselling, interpersonal psychotherapy, and social support [[105], [106], [107]]. Social support may include support from a partner, family members, friends, support groups, healthcare team members, online, and within religious and various other communities in which a patient is embedded [[108], [109], [110]]. Importantly, perceived partner support, rather than mere presence of a partner, is a key determinant of postpartum mental health [104]. For patients with SLE, adequate social support may buffer the added stress of managing a chronic illness alongside the challenges of new parenthood.

### Social support specific to SLE patients

Patients with moderate-to-severe SLE suffer from a substantial disease burden and require a high level of support from the community and their healthcare team. Many of the common symptoms experienced before and after diagnosis, fatigue, muscle and joint pain, poor sleep, and brain fog, contribute to a significant reduction in the quality of life for SLE patients [111]. Black women make up a disproportionate percentage of SLE patients and are at higher risk for experiencing increased morbidity and mortality throughout their disease course, most likely due to a combination of racial healthcare disparities and socioeconomic inequality [112]. Poverty, in particular, has been shown to significantly increase all-cause mortality in SLE [112]. During the peripartum period, the healthcare and socioeconomic burden on patients with SLE increases as, in addition to managing their autoimmune disease, they must receive prenatal care, cope with physiological changes to their bodies during pregnancy, and pay for the myriad expenses that come with caring for a newborn infant. The expected reduction in postpartum sleep quantity may also be compounded by a high level of baseline fatigue in lupus. Social support during this turbulent period is likely to have a positive impact, though studies of specific support interventions in patients with lupus are lacking.

### Strategies to improve social support

Given the interplay between social support, postpartum maternal health outcomes, and neonatal health outcomes, strategies to bolster social support should be utilised by healthcare providers. The first step in addressing gaps in social support of patients is becoming comfortable with asking patients about their social challenges in a caring, culturally sensitive, and respectful manner. Another way in which providers can facilitate such support is through ‘social prescribing’—the process of becoming familiar with the support services that are available to patients and acting as a reliable resource during the process [113]. This includes support groups, parenting classes, and local community and hospital support services. In addition, healthcare providers have the skills to facilitate family meetings to express the limitations and needs unique to postpartum patients with SLE in order to rally support in a concrete and tailored way. Finally, providers can ensure continuity of care and connect patients with home health visits to address problems promptly.

## CONCLUSION

SLE has numerous clinical and psychosocial effects on the postpartum period for birthing parents and infants. Among the topics reviewed, research into safe breastfeeding with concomitant disease control is the most developed, with recent guidelines from the American College of Rheumatology providing a foundation for shared clinical decision-making. However, significant gaps remain in understanding postpartum depression, parent-infant bonding, social support, microbiome changes, and the optimal treatment of lupus flares during this critical period.

The postpartum period is an essential time for fostering parent-infant bonding, which should be prioritised to support the infant’s long-term development. To achieve this, evidence-based interventions must mitigate the unique challenges posed by SLE. Current postpartum care should emphasise key strategies identified in this review, including adherence to medications for flare prevention, prophylaxis for thrombotic events, education on the safety of breastfeeding with immunomodulatory treatments, postpartum depression screening, optimisation of the perinatal microbiome, and strengthening social support networks.

This review underscores the urgent need for further research to address these knowledge gaps, with the ultimate goal of improving maternal and neonatal outcomes. By aligning clinical care with emerging evidence, healthcare providers can help ensure that the postpartum period is a time of connection, healing, and growth for SLE patients and their families.

## Funding

This research did not receive any specific grant from funding agencies in the public, commercial, or not-for-profit sectors.

## CRediT authorship contribution statement

**Erika G Fleming:** Writing – review & editing, Writing – original draft, Conceptualization. **Jamie Chen:** Writing – review & editing, Writing – original draft, Conceptualization. **Sarah Mohideen:** Writing – review & editing, Writing – original draft. **Anna Broder:** Writing – review & editing, Writing – original draft. **Antonia Francis Oladipo:** Writing – review & editing, Writing – original draft, Conceptualization.

## Competing interests

All authors declare they have no competing interests.
